# Perspectives and Beliefs Surrounding Postpartum Care and Physical Activity of South Asian Immigrant Women in Canada

**DOI:** 10.1177/08445621251395348

**Published:** 2025-12-09

**Authors:** Iris Lesser, Corliss Bean, Harleen Sangha, Bushra Mahmood, Scott Lear

**Affiliations:** 1 Faculty of Health Science, 210534 University of the Fraser Valley, Chilliwack, Canada; 2 Faculty of Applied Health Sciences, 7497 Brock University, St. Catharines, Canada; 3 Population and Public Health, BC Centre for Disease Control, 8166 University of British Columbia, Vancouver, Canada; 4 Faculty of Health Sciences, 1763 Simon Fraser University, Burnaby, Canada; 5 Centre for Heart Lung Innovation, St. Paul's Hospital, Vancouver, Canada

**Keywords:** immigrant, exercise, qualitative, maternity, mothers

## Abstract

**Introduction:**

Beginning or returning to physical activity is an important element of postpartum care due to its associated mental and physical health benefits. South Asian immigrant women often report a lack of confidence in physical activity engagement and may face cultural barriers to engagement. The purpose of this study was to explore South Asian immigrant women's perspectives and beliefs surrounding postpartum care and physical activity in Canada.

**Method:**

Eleven South Asian immigrant women (*M_age_* = 31) who had given birth in Canada within the last 12 months and were able to understand and speak English or Punjabi, participated in the study. A semi-structured interview was used to explore participants’ birth and postpartum healthcare experiences, as well as their physical activity engagement. Audio-recorded interviews were directly translated and transcribed from Punjabi to English and a reflexive thematic analysis approach was applied.

**Results:**

Through our analysis, we developed four themes: (a) conflicting traditional and modernized beliefs around postpartum care; (b) physical activity was important to care for one's child and the home, but perceptions of what constituted physical activity varied; (c) lack of time, lack of knowledge, a physically weak body, and exhaustion were barriers to physical activity engagement; and (d) social support was crucial in improving well-being and reducing feelings of isolation, particularly for those with live-in family.

**Conclusion:**

Given the high appreciation for community programming which support South Asian mothers, it is essential that culturally relevant physical activity be incorporated into healthcare education postpartum to increase knowledge around health benefits of physical activity. In addition, South Asian immigrant women may require community engagement (including programming) and informational support from healthcare providers.

Physical activity levels continue to decline globally with noted disparities across regions and sex, with females engaging in less physical activity than males (Strain et al., 2024). Physical activity levels in women tend to decline throughout motherhood (Albright et al., 2006), making the postpartum period (i.e., 12-months following the birth of a child) an important timepoint to promote physical activity (World Health Organization, 2022). However, physical activity promotion postpartum is broadly aligned with Western values and lacks cultural consideration. These considerations are crucial as promotion of physical activity must align with one's cultural traditions, values, and beliefs (Ramos, 2004), while also considering social contexts that influence participation. Understanding the culturally relevant context surrounding physical activity allows for more appropriate promotion of these behaviors, especially in minority populations (Rio & Saligan, 2023).

In Canada, immigration from South Asia (predominately from India) has increased fourfold from 1996 to 2021 to over 2.5 million (Statistics Canada, 2024). Inequities in maternal health care experiences and outcomes are seen in immigrant women (Fair et al., 2020; Higginbottom et al., 2015) with lower healthcare utilization and follow-up postpartum (Khanlou et al., 2017; Sword et al., 2006). In addition to direct healthcare access, South Asian immigrant women face many challenges including social isolation, family separation, socioeconomic hardship, discrimination, language barriers, and challenges navigating the healthcare system (Nagesh et al., 2024; Nilaweera et al., 2014) which impact their postpartum healthcare and well-being. Incongruence between traditional cultural practices and host country practices postpartum (i.e., newborn bracelets, prayer, head shaving, feeding infants ghutti or honey (Nagesh et al., 2024)) have further led South Asian immigrant mothers to feel conflicted with traditional practices due to a lack of cultural understanding by their host country and healthcare providers (Nilaweera et al., 2014). For instance, in India, South Asian women engage in the traditional practice of rest and recovery for the first 40 days postpartum (Sharma et al., 2016). Moreover, South Asian cultures place particular importance on the family unit during the postpartum period, which may impact experiences during this time such as seeking postpartum support from family members before reaching out to healthcare professionals (Goyal et al., 2015; Kandasamy et al., 2020). To reduce conflict and improve trust between patients and healthcare providers, culturally appropriate care and acknowledgement of the legitimacy and importance of traditional maternal practices is essential (Nagesh et al., 2024).

An important element of postpartum care is beginning or returning to physical activity given associated mental and physical health benefits (American College of Obstetrics and Gynecology, 2020). Among South Asian women, there are additional benefits of engaging in physical activity postpartum given this population's higher rates of postpartum depression and type 2 diabetes (Jenum et al., 2019; Narayan et al., 2021). Recently, the Canadian Society of Exercise Physiology released physical activity guidelines highlighting the importance of movement in the early postpartum period (Davenport et al., 2025). However, barriers to engagement are high among the broader Western population (Saligheh et al., 2016).

Among South Asian immigrant women, physical activity engagement is low, with only 17% meeting the physical activity guidelines of 150 min of moderate-to-vigourous physical activity per week compared to 36% of European women (Hayes et al., 2002). Despite the benefits of physical activity, South Asian immigrant women are largely physically inactive with noted barriers including work, culture, cost, time, family, health status, and environmental factors (Pullia et al., 2022). South Asian immigrant women often report a lack of confidence in physical activity engagement (Pullia et al., 2022) and pregnant South Asian immigrant women have reported negative views around physical activity engagement (Greenhalgh et al., 2015). Further, in a multi-ethic study, South Asian immigrant women had the lowest moderate-to- vigourous physical activity postpartum with Western women accumulating 26 more minutes of physical activity per day (Richardsen et al., 2016).

It is unknown how physical activity is approached after the birth of a child when the demands placed on South Asian woman increase in motherhood. For instance, the traditional values of a live-in support system and 40-day rest period, an understanding and confidence in beginning or returning to physical activity (when it is considered generally safe and beneficial for women to do so) may be impacted. With the extensive role of healthcare practitioners (i.e., nurses) in providing postpartum care, it is essential to understand the necessary support for encouraging physical activity postpartum among South Asian immigrant women. Therefore, the purpose of this study was to explore the perspectives and beliefs of South Asian immigrant mothers surrounding postpartum care in Canada, postpartum well-being and physical activity meaning and engagement postpartum.

## Methods

### Philosophical Approach and Methodology

An exploratory qualitative research study was conducted guided by a relativist ontology and a social constructionist epistemology. A relativist ontology posits that reality is subjective and aims to capture the richness of human experience by emphasizing that multiple realities co-exist (Pretorius, 2024). In line with this, a social constructivist perspective recognizes that knowledge accrued is an interrelationship between both participants and researchers as knowledge is shaped by interactions, dialogue, and personal narratives (Jones & Coast, 2013). As demonstrated in previous research with mothers and their physical activity experiences (e.g., Bean et al., 2023; Lesser, Bean, et al., 2024; Lesser, Wurz, et al., 2024; Ritondo et al., 2023), the current study was guided by social constructivism as it allows for an understanding of participants’ stories through both a personal and broader social lens which acknowledges the complexity of participants’ lived experiences (Sparkes & Smith, 2013).

### Positionalities

In our approach to qualitative research, we acknowledge the importance of reflexivity as our own identities and lived experiences frame the research process from conceptualization of the research through data collection and data analysis (Yoon & Uliassi, 2022). Therefore, we frame our personal experiences and perspectives to ensure transparency in our analytic approach (Palaganas et al., 2017). The first author (A1) identifies as a white able-bodied heterosexual third-generation woman, who is an Associate Professor in a kinesiology department at a Canadian university, is the mother of two young children and is active daily. The second author (A2) identifies as a white able-bodied heterosexual third-generation Canadian woman, who is an Associate Professor in a health sciences department at a Canadian university and is a mother to two young children. She is a former varsity athlete and being active is a critical part of her identity. The third author (A3) identifies as a South Asian able-bodied heterosexual second-generation woman who is an undergraduate student in a kinesiology department at a Canadian University and is passionate about health as well as her community. The fourth author identifies as an able-bodied, first generation South Asian immigrant women, who is a postdoctoral fellow at a Public Health institution and is a mother of one young child. The fifth author identifies as a man white able-bodied heterosexual third-generation Canadian man of two children who is active daily.

### Participants

Following approval by the University of the Fraser Valley’s Human Research Ethics Board (REB 101681), prospective participants were recruited through the South Asian Best for Babies program in Abbotsford, British Columbia, Canada either in person at a local temple or through an online workshop. The South Asian Best for Babies program is a free community program funded by the Public Health Agency of Canada that offers maternal and infant support through pregnancy and postpartum and is offered in Punjabi. Participants were eligible to participate in the study if they were less than 12 months postpartum and able to understand and speak English or Punjabi. Participants gave online informed consent through Survey Monkey when conducting a demographic survey with the research assistant at the time of their interview.

Eleven participants (all who had emigrated from India) voluntarily participated in the study (M*_age_* = 31; *SD* = 3.8; see Table 1 for demogtaphic information). The average age of participants' most recent child was 22 weeks (*SD* = 14.5), ranging from 4 to 51 weeks. For eight women, this was their first child, for two their second child, and for one participant this was their third child. Delivery modalities included an emergency Cesarean birth (*n* = 3), a planned cesarean birth (*n* = 4), and a vaginal birth (*n* = 4). Of the 11 participants, six had completed post-secondary education with three reporting that they had completed graduate school. Five individuals reported having two adults living in their household at the time of interview (themselves and a spouse), while six participants reported having additional adults living in the household (3 to 5). Most participants expressed that they would prefer to receive health information in Punjabi, with two preferring English.

### Data Collection Procedures

Participants provided the following information in the online survey with A3: age, country of birth, preferred language for receiving health information, the highest level of education attained, number of children, number of adults living in the household, pregnancy risk factors, type of birth (vaginal, planned surgical, emergency surgical), and age of recent child in weeks. In addition, participants were asked whether they met the physical activity guidelines of 150 min of moderate-to-vigourous physical activity per week at the time of interview. It should be noted that a definition of physical activity had to be provided for participants to respond to this question, as there is not a direct translation of physical activity into Punjabi, which was simplified from “any bodily movement produced by the skeletal muscles that result in an increase in energy expenditure” (Caspersen et al., 1985) to ‘movement of your body which increases your heart rate from resting’.

Following completion of the online informed consent and demographic survey, participants then engaged in a one-on-one semi-structured interview in Punjabi (preferred by all participants over English) with a woman-identifying female South Asian research assistant (A3 and a secondary research assistant). All interviews were conducted via Zoom and participants were asked to be in a quiet and comfortable environment during the interview, if feasible. Prior to beginning the interview, participants were informed of confidentiality of their data and that their data would be audio-recorded and then deleted after transcription. Participants were also informed that their statement would be anonymous and published using pseudonyms. Each semi-structured interview was divided into three sections. The first section included exploring participants’ birth and healthcare experiences postpartum and understanding whether these experiences would have been different in their birth country (e.g., “What can you tell us about how your postpartum experience here is different than in your country of birth?”). The second section included how participants felt about their own well-being since having their child (e.g., “What role did your healthcare provider play in how you felt about how to take care of your own well-being postpartum?). The third section explored physical activity meaning and engagement postpartum (e.g., “In your opinion, is it important for a mother to be physically active after the birth of their child?”; see Supplemental File 1 for interview guide).

### Data Analysis Procedures

Audio-recorded interviews were conducted in Punjabi and directly translated and transcribed from Punjabi to English by A3 and a secondary research assistant (note that not all Punjabi words have a direct translation to English). Braun and Clarke's (2019) reflexive thematic analysis approach was applied whereby we acknowledged our role as researchers in the generated knowledge created due to our own perspectives and lived experiences (Hesse-Bibber & Piatelli, 2012). This included A1 reading and re-reading the transcription to gain an understanding of the data. Data was then grouped into similar codes with further development of themes and subthemes using Microsoft excel. This analysis was then reviewed by A3 who conducted most of the interviews (*n* = 9), themes and subthemes were discussed in person with A1 and themes and subthemes were modified until there was agreement using an inductive research approach. The themes and subthemes were put into a table that was then analyzed and reviewed by the research team (A1, A2 and A3), who have expertise in postpartum physical activity, South Asian health, and qualitative analyses. The research team had the opportunity to review the data independently, meet, and collaboratively discuss and revise on several occasions. During these interactions, the data were discussed and re-interpretated until all individuals agreed upon the themes and subthemes and felt that there was an appropriate alignment (i.e., renaming or reconsidering themes and subthemes). To enhance the reading of the data, filler words (e.g., ‘like’, ‘uhm’) were removed and culturally appropriate pseudonyms were assigned to ensure participants’ anonymity.

## Results

Through our analysis, we identified four themes detailing participants’ experiences of postpartum care including traditional and modernized beliefs, the importance of physical activity in caring for one's child and home, the challenges of physical activity engagement postpartum, and the importance of social support postpartum in improving well-being (see Table 2).

**Table 1.** Participant Demographics and Maternal History.

Age of Mother; M(SD)	31 (3.8)
Number of Children	
First Child	8 (72.8%)
Second Child	2 (18.2%)
Third Child	1 (9.10)
Age of Child	22 (14.5)
Birth modality	
Emergency Cesarean	3 (27.3%)
Planned Cesarean	4 (36.4%)
Vaginal	4 (36.4%)
Perinatal Risk Factors	
None	8 (72.8%)
Anxiety	2 (19.2%)
Gestational Diabetes	1 (9.10%)
Education	
High School	1 (9.10%)
Post Secondary	7 (63.6%)
Graduate School	3 (27.3%)
Adults in Household	
Two	5 (45.5%)
Three	1 (9.10%)
Four	4 (36.4%)
Five	1 (9.10%)
Preferred Language	
English	2 (19.2%)
Punjabi	9 (81.8%)
Meeting Physical Activity Guidelines (150 min. of moderate-vigourous physical activity/week)	
Yes	2 (19.2%)
No	9 (81.8%)

**Table 2.** Summary of Themes and Subthemes.

Themes	Sub-theme(s)
Conflicting traditional and modernized beliefs surrounding postpartum care	Modernized perceptions of motherhood
Physical activity was important to care for one's child and the home, but perceptions of what constituted physical activity varied	Being active meant not being inactive Being active *with* and/or *for* baby If not a household duty, then we walk
Lack of time, lack of knowledge, a physically weak body, and exhaustion were barriers to physical activity engagement	Dependency on online physical activity information
Social support was crucial in improving well-being and reducing feelings of isolation, particularly for those with live-in family	Tension between prioritizing infant caregiving over self-care

### Conflicting Traditional and Modernized Beliefs Surrounding Postpartum Care

Mothers discussed how postpartum care would be different if they had given birth in India as cultural values would ensure a period of rest and recovery. Postpartum care practices in India often include the support of a *japa* who is hired to support maternal care and recovery with holistic massage. As described by Praveen who had her first child in India: “In India, I rested for two whole months and for 45 days, a lady used to come to our house and properly massage me every day.” The importance of rest and maternal care postpartum was noted from both traditional Indian practices and modern practices in Canada amongst participants who had live-in family support. Anita stated:

We are going through rebirth, so we need to properly take care of ourselves like watching how we eat, walking, and for those (with) C-section, they have a harder time because their body has been cut, so a mom needs as much care as the baby.

Mothers described this traditional model as allowing for faster recovery from birth by following a range of cultural practices postpartum. Seema described what traditional postpartum care practices are in India: “They’ll tell you to cover your head when you go out, put socks on your feet, don’t go outside, don’t go in the wind, your joints will have pain.” There were many discussions around diet and traditional beliefs of what to eat to support postpartum recovery or to support lactation, including avoiding spicy food, drinking warm water and avoiding ice water, and consuming *panjiri* (a sweet snack made from nuts and clarified butter) as it is thought to assist in recovery after giving birth: “You should eat *panjiri* and this will make your body strong” (Seema). Some of these traditional practices differed from what was discussed by healthcare practitioners in Canada (modern beliefs), as described by Praveen: *“*In India, they tell you don’t eat this, they told me if I eat something, the baby will have gas. But here, they tell me I can eat whatever I want.”

For some mothers, following a non-traditional model of postpartum care was found to yield greater independence. Providing care of their infant without additional family support or advice was discussed as enhancing their confidence postpartum. Preeti, who did not have live-in family support and engaged in more modernized beliefs stated:

If I was [in India], I would have gained more weight because I would have rested only, but here I got more experience taking care of the baby, I'm doing everything independently––taking care of myself and the baby. In India, I would have felt everyone is here to look after me. So I would have gotten more lazy, but I wouldn't have to struggle as much there since I would have more help.

Similarly, Jyoti outlined:

[In India], you don't have to leave your house because your family's there to support you so you are mentally relieved. But then there's also other pressure, from [Indian] society that you should do this or shouldn't do this to your baby. Here, I take care of him myself, look at what time he should nap, what time he should eat, so he has a routine here that he might not have in India.

#### Modernized Perceptions of Motherhood

Participants discussed how postpartum care would have differed if they had their child(ren) in India as family and cultural values are more traditional whereas having their child in Canada allowed for more modernized perceptions of motherhood, as discussed by Rekha: “People in India have a different, more traditional way of thinking.” This more traditional Indian way of thinking is described as an inability of new mothers’ parents or in-laws to understand the perspectives of immigrant mothers postpartum. “Parents have a habit of saying stuff like that since they’re from the older times where even if we tell them about mental stress, they’ll say ‘what's that? That didn’t happen to us, we had kids too” (Jyoti). Participants often turned to their physician or the internet for information postpartum, but this created a generational divide with more traditional family views when following more modernized beliefs, as noted by Rekha:

We listen according to the doctor, and we search things on Google ourselves but if we tell our family members, they will disagree and say that they raised kids in a different way but that was an older time. I think that there are two different generations, and that gap affects it a lot.

These more traditional viewpoints from India can lead to mothers feeling judged if they follow more modern beliefs in Canada around taking care of oneself and engaging in physical activity: “Some older generations have that thinking like she doesn’t stay home with the baby, she just thinks about herself and wants to have her body slim” (Jyoti). Some mothers also discussed the pressures of fulfilling their ‘gendered roles’ in tending to household chores. These patriarchal views were discussed by participants as relating to the traditional Indian view that it is a woman's responsibility to engage in household duties, such as cooking, cleaning, and childcare: “In our culture they’ll say men don’t do housework, girls do more of housework, so that's why we, ladies, feel more pressure to do all the work” (Seema).

Mothers further described how these traditional beliefs were less prevalent for them, either as being part of a younger generation or due to living in Canada where there are more modern parenting views, as discussed by Puthija:

People born in India think from an Indian's perspective and approach parenting in a similar way, but people born here [Canada] live their life exactly the way they are taught to live in Canada following Canadian traditions and care for their babies and protect them in a more Canadian way.

Living and raising their children in Canada allowed them to embrace more modern parenting values, as shared by Jyoti:

In Canada, we can raise the baby according to ourselves and we don’t have to worry about what others say about what we should or should not give the baby. Here, we are more educated on what is important, what the baby needs, and what we should be doing according to today's knowledge. If my baby was [in India], he would be raised according to how it is in India. Here (Canada), I am going according to the new times here.

### Physical Activity was Important to Care for One's Child and the Home, but Perceptions of What Constituted Physical Activity Varied

Physical activity was often seen as embedded within daily tasks and included household duties and childcare tasks. Mothers who felt that physical activity was defined within these duties rated their physical activity levels higher than mothers who felt that physical activity was independent of these responsibilities. Those who engaged in physical activity outside of these responsibilities preferred outdoor walking or sought online programming that typically took place with their baby present. Moreover, there was a common thread that mothers’ physical activity was important to care for the child, as outlined by Rekha and Seema:

Physical activity is looking after our health or staying in shape since we gain[ed] a lot more fat after pregnancy. According to me, exercise is walking and that’s what I think is being physically active… it’s very important to be active. If we are active, then we can look after the baby too. (Rekha)

Being active means doing everything and managing it, and when you are active, your body will feel it too. I will also exercise when the weather is nice out and from that, my body feels relaxed and the baby will also feel relaxed. If my health and my mind is good, then my baby will be happy too. Only then, I can take care of her too if I am active myself. If I was lazy and didn’t get anything done, then how will I take care of baby? (Seema)

#### Being Active Meant Not Being Inactive

When asked about their physical activity engagement, many women discussed how being active meant not being inactive. Since physical activity was often viewed as getting out of bed and completing household duties, many women rated their current physical activity level as high. For Praveen, physical activity was defined as the following: “Don’t sit in your bed all day […] do anything, go for walks, but just don’t make yourself lazy.” … Being physically active is very important for the baby and for yourself. Now that the baby is born, we shouldn't stop taking care of ourselves either. We should prioritize ourselves.”

Preeti described physical activity as “Where we have movement in our body. We can say housework, going out for walks, if we do any work in the house…this is what physical activity is referring to.” Physical activity engagement postpartum varied among participants, partially dependent on how recently they have given birth. For instance, when describing her daily routine, Anita discussed how many of her parenting and household duties keep her active:

The first hour is doing work in the kitchen, we’re active here, and then for 30–45 min, we go walk to the park so we’re active then, and 15–20 min, I’ll do some exercise for myself so I am active here, and then for another 30–45 min, I will play with toys with my daughter so I am active then too.

These daily household duties were described as quite vigorous by Preeti: “My day goes by with me running around…going up the stairs and then down the stairs like my life just consists of activities. I get tired, my legs start hurting.” These household duties were noted to be harder work when compared to before having a baby as there was less time now to complete tasks and therefore, these duties were done quickly and more intensely. Women who found household duties to be time-consuming and more vigorous typically rated their physical activity levels as high: “By living alone, I'm managing everything—my baby, household duties, and even studying—while staying active” (Preeti). This diverged from participants who did not consider being active around the home as physical activity who rated their physical activity engagement much lower. Praveen discussed her low physical activity engagement as: “Most of the time I just feel like I didn’t even do anything, just housework. I made food, I made my way to the kitchen and back with the baby, that's all for now. I don’t get any other activity.” Therefore, physical activity independent of household duties and childrearing was a challenge for most participants.

#### Being Active with and/or for Baby

Participants described physical activity as including their baby or expressing that the reason they engage in physical activity is to take care of their baby and the associated household duties. As Suman and Seema simply stated: “If a mother is active, she can properly take care of the baby” and “we need good well-being since we have to take care of the baby. We need to be strong for the baby”. Incorporating one's baby into their routines through stroller walking or by exercising with their baby nearby were common physical activity practices, particularly among mothers who did not have live in social support. Priyanka participated in physical activity with her baby but described challenges of trying to follow programming:

I’ll put the baby on the swing and then do exercises next to him, but I can’t continue. Like it's not like I can do 20 min straight…I have to take breaks and catch my breath then slowly do it. I get tired.

If their baby was not incorporated into their physical activity routine, mothers described waiting for naptime or for someone to watch their child so they could engage in physical activity, as Preeti noted: “I have to do all the activities while he's sleeping in a short time. I have to do all the work fast now because I feel like I should get all the work done before he wakes up.” Moreover, Puja described being more independent as increasing her physical activity levels: “I think I'm more active after having the baby because I now manage many things—work, household duties, and taking care of the baby and doing the activities with him.” Participants also discussed learning to be flexible with their physical activity engagement, as Rekha described what fitting in her walking looked like each day: “The timing is just not consistent…I don’t do 30 min every day. It's not a certain time in a day either, it's just whenever I get time.”

#### If not a Household Duty, Then We Walk

Women who expressed engagement in physical activity outside of their household duties discussed walking as their physical activity. Specifically, Preeti outlined: “[Physical activity] is where we have movement in our body. We can say housework, going out for walks, if we do any work in the house, this is movement, this is what physical activity is referring to”. Walking outdoors was consistently discussed as only being done when the weather was dry and warm. Praveen notes that: “Because of the weather, we have been stuck at home too but now the weather's getting better so I think I will start to take him out for walks with me.” Priyanka, who had older children, described how cold weather kept them all inside:

If the weather is nice in the afternoon, then I take the kids with me while they ride the bike, and they’ll walk with me…I put the little one in the stroller. But the weather isn’t that nice so since it's cold, we don’t go that much.

This lower physical activity engagement was discussed in comparison to their physical activity levels before having their infant, when many described engaging in high levels of occupational or transportation-related physical activity, such as walking for work or commuting. They noted that this form of activity was no longer part of their routine, and even leaving the house had become a challenge. Participants described this as a stark contrast to their experiences in India, where they had more space to move throughout the day, allowing them to be more active with their baby. Conversely, in Canada, participants felt more confined within their homes. Praveen reflected on this difference between her home in India and her experience in Canada:

In India, everything is a lot more open spaced. Here we live in a basement; it's just from here to one room, to another room, that's it. But in India, you go upstairs, downstairs, outside, there's a lot more space for activity. You just naturally get more steps in.

### Lack of Time, Lack of Knowledge, a Physically Weak Body, and Exhaustion Were Barriers to Physical Activity Engagement

Barriers to physical activity engagement among this sample included a lack of knowledge, feeling that their bodies were weak, exhaustion, and a lack of time. Due to time constraints, Suman found it difficult to engage in physical activity due to discomfort from milk leakage, while Priyanka described physical challenges that felt beyond her control: “It was really hard to lift my leg up or put it back down.” Similarly, Praveen shared: “I just feel like my body isn’t working,” which demonstrates the barriers of feeling weak and exhaustion.

Lack of knowledge was another seen as another barrier to engaging in physical activity. While most women shared that they felt heard, well-informed, and understood by their maternity care providers, some described feeling uninformed or noted that language barriers made it difficult to feel confident in their knowledge. Participants discussed the high importance of community programs (i.e., Best for Babies), which provided infant and maternal care in Punjabi, as a place of support and education that was vital, particularly to those without traditional social support.

Women who did not view household activities as part of being physically active described how attempting to engage in physical activity outside of these responsibilities was overwhelming and greater social support was needed to overcome barriers, as stated by Pinki: “The biggest challenge is that I don’t have time to do anything. I must do the household duties faster than before giving birth…everything is on my own, and that's why I can’t really focus on physical activity right now.*”* This challenge in finding time to engage in physical activity due to a lack of support was further discussed as being unique to living in Canada where there is less of a village to support raising children: “Here in Canada, it's a big problem. The mother doesn’t get enough time for herself. If we are healthy, then only our kids will be healthy.” Therefore, a lack of support for mothers to have adequate time for physical activity engagement was a challenge for South Asian immigrant mothers without social support.

Despite some optimistic perspectives around navigating physical activity postpartum, some mothers were hard on themselves when they did not meet their physical activity goals and described feeling guilty, as Jyoti shared: “When I don’t go some days, I feel depressed with myself…I feel guilty like I didn't do any activity with my baby and that this is wrong of me as a mother.” Similarly, Priyanka stated how: “Some days, I feel like I didn’t do anything so then I feel bad, like ‘oh no I did nothing today.’” Therefore, there is a balance between physical activity promotion and the potential negative impact of further overloading new mothers.

#### Dependency on Online Physical Activity Information

Women expressed turning to the internet for other specific forms of physical activity guidance as they did not feel like they had received this information from their healthcare provider. As shared by Anita: “My husband would search what's right or wrong for a woman postpartum because I didn’t know how to maintain my body…after pregnancy. He would look at Instagram or snapchat, he’d look at reels and think this could be useful.” Rekha described how she was not told anything about physical activity postpartum so she sought information from the internet: “I just look on my phone on my own and learned that after six weeks, you can recover from pregnancy and can start to work out.” Further, Puja sought out specific exercises to target her waist after pregnancy: “I do simple exercises while lying on the floor, watching on the internet such as leg folding exercises to reduce my stomach and belly fat.” Therefore, participants who aimed to engage in physical activity postpartum largely relied on online sources.

While there may be benefits in accessing online information, it also yields negative social comparison, as Praveen described: “I see reels on Instagram and I see people online who start exercising right away even after C-section, I don’t know how they do it.” Accessing physical activity information online was often discussed in reference to weight maintenance or weight loss, as Anita stated: “If I felt like I gained too much weight, then I would exercise, that's all.” In addition, Rekha described how physical activity is only engaged in postpartum to improve one's shape given weight gain experience in pregnancy. Similarly, Preeti noted: “[If] we become lazy…everything stops. The baby will also be lazy…sleeping all day and us too…our body will get too fat. When we stay active, we stay a little bit fit.” For Rekha, weight retention was causing her mental duress:

I would say my biggest problem is my looks…because my weight is quite high. I check it every week but it's not going down, I am trying a lot and doing what I can. So, this upsets me when I wear a dress and clothes don’t fit me. I think to myself if I will ever fit into them again, so this is mental stress.

### Social Support was Crucial in Supporting and/or Improving Well-Being and Reducing Feelings of Isolation, Particularly for Those with Live-in Family

Whether it was live-in family support, husbands, or friends, women emphasized the importance of having someone to support their mental well-being through postpartum. Many participants described how they had live-in social support and felt cared for through their postpartum recovery, while others, who did not have this live-in social support, emphasized the importance of their husbands or created their own community to navigate this phase. Jyoti, who was living with roommates while her family, including her husband, were still in India, described the importance of creating her own community: “If they are positive and they support you like, ‘sit down and eat, shower, don’t worry, we’ll hold the baby’, this helps a lot and keeps you positive.” Those who did not have a traditional model of live-in family support largely reported having husbands who were able to provide adequate support. Seema described that her husband supported household tasks and with their infant:

I didn’t have to do any work around the house; everything was taken care of. My husband would bathe the baby, and with making roti, he would help. He would also make food and give milk to the baby; he did it all. When the baby was crying, he would pick her up, and I would get some rest.

It is worth noting that there were some challenges of social support despite its many benefits due to conflicting viewpoints around parenting, as stated by Rekha:

My family will be like no you have to feed her, pick her up or quiet her down…so I feel pressure during this. I don’t want to usually…I’ll think I just fed her…why is she crying? So, then my family will just say “No, no feed her” even though I’ll say no, I did feed her” denoting the challenge of navigating advice.

As such, there were mixed perspectives around the value of live-in family support despite its many advantages outlined. Further, while partner-based support was recognized as important, it was also described as not being adequate when compared to the level of support one would receive from ones extended family and community in India, as noted by Sangit.

In Canada, there's no cultural or social norm for physical activity postpartum. It's all about personal choice. My husband helps sometimes, but other than that, there's no support from extended family or neighbors. In Canada unlike India, we cannot count on our neighbours.

Those who did not have this support seemed critical of the Canadian model which emphasized having to do it all themselves. They struggled with postpartum responsibilities, feeling that additional support would help in physical recovery from birth, as well as in balancing household responsibilities, and managing mental well-being. Sangit described the benefit of having live in family care in enhancing her mental well-being: “I didn’t feel overwhelmed because my husband and everyone would take care of me.”

Concern around mental well-being postpartum was evident among South Asian immigrant mothers with many reporting fear that they were becoming depressed. For some women, this fear around postpartum depression was due to not having anyone to take care of their child if they were not well. Jyoti shared: “It was my first baby and there were no family members with us. I used to think ‘will I be able to do it’?” For other women this concern around developing postpartum depression was due to others sharing their own experiences: “Other girls would talk and say that after having kids, you have depression. I don’t know if it's because I was hearing this and that's why I started feeling like what if I get depressed” (Priyanka). This fear of postpartum depression was vastly recognized, as many mothers described emotional upset in the first few days after birth, which faded after this early phase of recovery: “Mentally, I would get depressed sometimes because I am a first-time mom so when the baby was newborn—two or three days old— when she would start crying a lot, I would worry about why she is crying” (Rekha).

#### Tension Between Prioritizing Infant Caregiving Over Self-Care

Women reported internal conflict when engaging in physical activity in the face of infant care responsibilities. Broadly, women reported the importance of prioritizing their own physical activity to be able to be active for their child: “I feel that now that the baby is born, we shouldn’t stop taking care of ourselves either. We should prioritize ourselves.” This was discussed as being common among South Asian culture, particularly what was described as newer mindsets. Physical activity was also seen as important to be able to be in good health and care for family members, as discussed by Rekha and Jyoti, respectively: “If you focus on your health, it can go back to how it was before and with that, we’ll be more active with our baby and give them more of our focus” and “If you are healthy, you can do more activities, spend more time with them and your baby will also be healthy as you keep them active playing with them and involve them in more activities.”

## Discussion

The purpose of this study was to explore the perspectives and beliefs surrounding postpartum care and physical activity of South Asian immigrant women in Canada. The postpartum period is important to prioritize physical activity to support women's health and well-being, yet is understudied in minority populations, particularly South Asian immigrant woman. This study provides context as to the experiences of South Asian immigrant woman as they navigate postpartum care and physical activity engagement in a Canadian context. Our findings reveal conflicting traditional and modernized beliefs around postpartum care, varied perceptions of physical activity and physical activity postpartum, challenges around physical activity engagement and the importance of social support in enhancing physical activity and well-being.

Cultural differences in postpartum care practices between participants birth country of India and birth experience in Canada conflicted. Many participants noted that traditional models of postpartum care aided in maternal recovery but were either not practiced in Canada due to modernized medical views, or a lack of live-in support made maternal care impractical. Previous work has found challenges of healthcare views of South Asian immigrant women which did not align with modern medical practice in Canada (Nagesh et al., 2024; Nilaweera et al., 2014), however, some mothers were found to appreciate a non-traditional model of postpartum care as allowing for empowerment and independence in motherhood. The importance of social support in improving mental well-being postpartum was frequently discussed by participants in the context of fear around experiencing postpartum depression and parallels what has been found amongst South Asian mothers in other studies (Jones & Coast, 2013). Mothers without this social support did discuss empowerment around their own learned independence despite the struggle aligning with the notion that learned coping is essential to postpartum health (Khademi & Kaveh, 2024).

Physical activity was denoted as important to care for one's child and the home, but perspectives around what constituted physical activity differed. South Asian immigrant women in this study described a large amount of responsibility in the home environment which they identified as physical activity. Women in South Asia bear the brunt of unpaid care work (Tripathi et al., 2022) and women in this study stated that this would be the expectation if they were in India. However, living in Canada, most participants did not feel that this was their responsibility from a cultural perspective. Despite this, a large amount of this work still falls to the mother due to childcare responsibilities and the need for food to be prepared. While household duties are often not viewed as exercise (i.e., planned, structured or repetitive), household activity is categorized as light-to-moderate physical activity (American College of Sports Medicine, 2025) and is associated with decreased mortality (Shi et al., 2024). This aligns with the statement that “something is better than nothing” when it comes to health and physical activity (Bull et al., 2020). South Asian immigrant women have been found to have differing opinions as to whether they believe household activities to be considered a form of exercise (Mahmood et al., 2022). While some of these household duties may be beneficial for physical health (Shi et al., 2024), it may be less impactful for mental well-being, where activity is demand-oriented rather than fostering enjoyment (i.e., leisure-based physical activity). White et al. (2017) found no association between household physical activity with mental well-being despite being positively associated with leisure-based physical activity. Given the importance of physical activity postpartum to support mental well-being (Davenport et al., 2025), it should be recognized that while all movement counts, not all movement may positively benefit mental well-being.

South Asian immigrant women in this study discussed the importance of social support, particularly live-in support, in managing their own health and well-being, as well as childcare and domestic tasks after the birth of their child. The live-in support (55% of participants reported three or more adults in the household) seemed to reduce the discussion around mom guilt (i.e., guilt mothers commonly experience taking care of their own needs in the context of mothering) which is common in Western populations (Bean et al., 2023; Bean & Wimbs, 2021; Lesser, Bean, et al., 2024; Lesser, Wurz, et al., 2024). In fact, mothers in this study discussed the importance of taking care of their own health and well-being to be able to take care of their child which deviates from the cultural view that if you are “skinny” you don’t need to exercise (Dave et al., 2015). This perspective aligns with postpartum care practices in South Asia focusing on weight management rather than physical activity as independent health behavior (Kaur et al., 2021). While most participants expressed having this social support, those who did not, described the overwhelming experience of attempting to “do it all” and did not find that there was time left to take care of their own well-being or to consider physical activity. This finding aligns with what has been previously found among postpartum women where a lack of time made physical activity unrealistic (Bean et al., 2023; Lesser, Bean, et al., 2024; Lesser, Wurz, et al., 2024; Ritondo et al., 2023).

Leisure-based physical activity discussed was primarily in the form of outdoor walking. Walking, particularly outdoors, is an easily accessible form of physical activity that provides many health benefits (Legrand et al., 2022). However, participants noted only walking for leisure when the weather was warm and sunny. Given the variable weather in Canada, this suggests low engagement a large portion of the year. Weather-related barriers are frequently cited as challenges with adherence to outdoor walking in Canada in diverse populations (Clark & Scott, 2016; Lesser et al., 2021). Moreover, knowledge around rehabilitation exercise postpartum was limited with women seeking health and physical activity information from family members, the internet and social media. Lack of knowledge surrounding physical activity postpartum has been previously reported in South Asian women (Babakus & Thompson, 2012). A small subset of participants who engaged in more structured exercise described seeking out postpartum physical activity information on social media, with the goal of reducing belly fat. Postpartum women have previously reported seeking physical activity information online (Ritondo et al., 2023). Given the South Asian cultural value of respect and trust in healthcare providers (e.g., physicians), further support around postpartum physical activity engagement would be valuable to reduce misinformation from online sources.

Participants in this study were recruited from community programming (i.e., Best for Babies) in support of South Asian mothers after giving birth. Content provided during programming included information about pregnancy, labor and delivery, parenting and nutrition with the intention of supporting maternal and infant well-being. Benefits of this recruitment include a homogeneous sample of participants who represent new South Asian immigrants to Canada, identify as South Asian, need maternal support, and prefer to receive health information in Punjabi. Limitations of this sampling approach include participant perspectives that may differ from those of other South Asian women who have been in Canada longer and not having information regarding socioeconomic status which may have been low despite education due to immigration. Strengths of this work include the delivery of interviews in Punjabi to promote inclusion and reduce language barriers. Potential limitations of translation into Punjabi and back into English include a lack of direct translation of certain terms such as ‘physical activity’ which can only be translated to ‘exercise’ and therefore required research assistant descriptive interpretation of some of the questions such as physical activity includes movement which increases your heart rate and makes you feel breathless. Future research should consider a more intensive description of participants’ immigration and socioeconomic status and a more diverse participant sample.

### Implications for Practice

South Asian immigrant women should be supported in engaging in physical activity intended to support their mental well-being rather than just being active through domestic duties. Given the high appreciation for community programming which support South Asian mothers, it is essential that physical activity be incorporated into healthcare education postpartum to increase knowledge around health benefits of physical activity. As postpartum South Asian immigrant women prefer walking as their primary form of physical activity, but engagement is weather dependent, opportunities for indoor walking or physical activity programming should be provided. In addition, women who immigrate and do not have live-in family support may require more community and healthcare support than those who have direct support. To ensure equitable healthcare support amongst immigrant mothers in Canada consideration of cultural beliefs postpartum should be understood and physical activity goals should be individualized. Recent postpartum physical activity guidelines (Davenport et al., 2025) should be further explored in relation to immigrant woman to ensure culturally aligned messaging.

## Supplemental Material

sj-docx-1-cjn-10.1177_08445621251395348 - Supplemental material for Perspectives and Beliefs Surrounding Postpartum Care and Physical Activity of South Asian Immigrant Women in Canada Supplemental material, sj-docx-1-cjn-10.1177_08445621251395348 for Perspectives and Beliefs Surrounding Postpartum Care and Physical Activity of South Asian Immigrant Women in Canada by Iris Lesser, Corliss Bean, Harleen Sangha, Bushra Mahmood and Scott Lear in Canadian Journal of Nursing Research
