# Supplementary material for: Perspectives and Beliefs Surrounding Postpartum Care and Physical Activity of South Asian Immigrant Women in Canada
Source: Can J Nurs Res. 2025 Dec 9;58(2):129–40. doi: 10.1177/08445621251395348 (PMC12989634; doi:10.1177/08445621251395348)
Supplement: sj-docx-1-cjn-10.1177_08445621251395348 - Supplemental material for Perspectives and Beliefs Surrounding Postpartum Care and Physical Activity of South Asian Immigrant Women in Canada [file sj-docx-1-cjn-10.1177_08445621251395348.docx]

**Beliefs and values around physical activity postpartum in immigrant South Asian mothers**

Hi, thank you for taking the time to be interviewed today. As you may recall, the purpose of these questions and interview is to understand your beliefs and values around physical activity since the birth of your child.

I have some information that I am going to share with you now just so you are clear as to what your participation involves. Feel free to interrupt me anytime if you need or want me to slow down or repeat anything.

You have been given the consent form where you have agreed to participate in this study. As a reminder, your responses and identity will be kept anonymous. As a member of the research team, I will know your identity, but I will assign a code to your name so that no one else from the research team can identify what you will share with me in the interview today.

Your participation is voluntary, and you can withdraw at any time and without any prejudice to you. There are no right or wrong answers. Your perspective will help us understand more about the experiences of South Asian moms moving more, and what may be useful to support South Asian mothers. Know that you can always skip a question if it makes you feel uncomfortable.

This interview will be audio recorded and used only for the purposes of data analysis by the research team. The audio recording will be stored in an encrypted database. Do you have any questions related to this interview? [if yes, interviewer to answer/address; if no, continue to next question.] Do you agree to participate in the interview? [if yes, continue; if no, terminate interview after asking if they have a reason and thanking them for their time].

Interview Guide

1. Can you describe your experience with your overall well-being since having your baby?

- How do you feel overall about your own well-being since the birth of your child? Do you feel as though you are in control of your well-being?

1. In what way would this experience have been different had you given birth in your country of origin? (for first generation immigrant women)

- Do you think these differences may have affected your health and well-being? How so?

1. What can you tell us about how this experience can be different for a South Asian woman (For those whose country of birth is Canada – or second-generation immigrant women)

- Do you think these differences may have affected your health and well-being? How so?

1. What is your personal belief regarding whether a new mother needs to focus on her own well-being after giving birth to a baby?

- How much of this belief has to do with the South Asian culture that you identify with?

1. Is your ability to focus on your own well being influenced by your family?

- Is this support from your family?
- Do you feel added pressure from your family?

1. What role did your health care provider play in how you felt (knowledge and support) about how to take care of your own well-being postpartum?

Giving birth is a life changing event in many aspects. It can impact our daily routine as well as our lifestyle behaviors like diet and physical activity. We will now ask you a few questions about physical activity and how it may have changed after the birth of your child.

| 1. What does the term physical activity mean to you? 2. What is your current physical activity like?  - Can you describe what a typical day looks like for you and what you would consider to be physical activity throughout that day?  1. How does your physical activity now compare to your physical activity before your child was born (compare to your pre pregnant self)?  - Were you more/less active before? - What changed? - What are your physical activity levels now – how many min of PA per day/what type of PA?  1. In your opinion, is it important for a mother to be physically active after the birth of her child?  - How soon after birth? - Why is it important? Or why not? |
| --- |
| 1. What are some challenges you have experienced with physical activity engagement since having your child?  - How has family impacted your physical activity engagement since having your child? - How have domestic duties impacted your physical activity engagement since having your child? - Do you think these challenges are due to social and cultural norms? - How has having other children impacted your physical activity engagement since having your child?  1. What has helped you be more physically active since having your child?  - How has family impacted your physical activity engagement since having your child? - Do you think these facilitators are due to social and cultural norms? |
| 1. With 1 being low and 10 being high, how satisfied are you with your physical activity level currently?  - Why would you say this? - Is there anything that would help to increase your rating?  1. Thank you for taking the time to participate in this study. Is there anything else about your experience postpartum that you would like to share with us? |
|  |
